# Effect of Ligustrazine on Endometrium Injury of Thin Endometrium Rats

**DOI:** 10.1155/2019/7161906

**Published:** 2019-06-11

**Authors:** Qing Ye, Yu Zhang, Jiulu Fu, Yi Zou, Wei Zhao, Chen Chen, Kun Liu

**Affiliations:** ^1^Department of Obstetrics and Gynecology, Affiliated Hospital of Nantong University, Nantong, China; ^2^Department of Traditional Chinese Medicine, Affiliated Hospital of Nantong University, Nantong, China; ^3^Department of Cardiothoracic Surgery, Affiliated Hospital of Nantong University, Nantong, China

## Abstract

The purpose of this experiment is to establish a rat model of thin endometrium and to explore the effect of ligustrazine on the thin endometrium of rats. The thin endometrium model was made by using infusing absolute ethyl alcohol into the uterine cavity. The thickness of endometrium was measured. Hematoxylin-Eosin (HE) staining was used to observe the histopathological changes of endometrium. The mRNA levels of VEGF, VEGFR-2, PI3K, and AKT were detected by RT-PCR. Western blotting was used to detect the levels of VEGF, VEGFR-2, PI3K, and AKT in endometrial tissue. The thickness of endometrium in the model group was significantly thinner than that in the control group. Compared with the model group, the thickness of endometrium in ligustrazine group was increased. HE staining shown that ligustrazine restored the histopathological changes of endometrium. RT-PCR and Western Blotting results showed that the mRNA and protein levels of VEGF, VEGFR-2, PI3K, and AKT in the model group were significantly decreased compared with the control group, while ligustrazine restored the changes. Ligustrazine can improve the morphology of endometrium, can promote the growth of endometrium, and has obvious therapeutic effect. Its mechanism is related to the activation of PI3K/Akt signaling pathway through upregulation of VEGF and VEGFR-2 expression to induce the repair of thin endometrium in rats.

## 1. Introduction

Thin endometrium means that the thickness of endometrium is lower than the threshold thickness for pregnancy. Specifically, it means that the endometrial thickness is less than 7 mm or 8 mm in the middle luteal phase (i.e., 6-10 days after ovulation), and its main clinical feature is the lack of menstrual flow without obvious reasons and it can lead to repeated abortion or infertility, resulting in a decline in the success rate of assisted reproduction, seriously affecting the quality of life of women. Miwa et al. [1] found that the main pathophysiological features of this disease were slow growth of glandular epithelium and high resistance to uterine artery blood flow. According to this characteristic, many researchers and clinicians have explored the pathogenesis of thin endometrium from many aspects and achieved some results. Aspirin is one of cyclooxygenase (COX) inhibitors, which can inhibit platelet synthesis and prevent microthrombosis. It can also inhibit uterine blood vessel contraction and reduce arterial resistance, thus improving intimal blood supply. Many studies have confirmed that [2–5] aspirin could increase endometrial thickness and improve endometrial receptivity. There is no clear cause for thin endometrium. Many scholars believe it is related to many times of uterine operation history, pelvic inflammatory diseases, uterine fibroids, uterine malformations, high blood flow resistance of uterine arteries, etc. More and more patients with menorrhagia and amenorrhea of unknown etiology in clinic show poor endometrium development and thin thickness during hysteroscopy. After treatment with exogenous sex hormones or endometrial stimulation, the effect is not good and they become potential infertility patients. Although the exact mechanism of thin endometrium is not clear, many studies in recent years have shown that too thin endometrium could significantly reduce the implantation rate of blastocysts. Basic studies have further confirmed the fact that the intrinsic relationship between thin endometrium and infertility lies in the decreased receptivity of endometrium [6]. At present, the main clinical treatments for thin endometrium include hormone therapy, drugs to improve blood flow perfusion, surgical treatment, G-CSF intrauterine perfusion, and regenerative medical stem cell therapy [7]. Although it is helpful to the treatment of thin endometrium, there are still many disputes about its safety and standardization. Therefore, based on the important relationship between thin endometrium and clinical pregnancy rate, how to effectively and safely improve the morphology and function of endometrium and increase the thickness of endometrium is the core issue to improve the clinical pregnancy rate.

Traditional Chinese Medicine has obvious advantages in treating female diseases. Ligustrazine is the first alkaloid extracted from the rhizome of Ligusticum chuanxiong Hort. In China, it is one of the effective components of Ligusticum chuanxiong Hort. in the treatment of cardiovascular and cerebrovascular diseases [8]. Pharmacological studies have proved that ligustrazine (TMP) has the functions of dilating blood vessels, increasing arterial blood flow, inhibiting platelet aggregation, and reducing platelet activity, and has the characteristics of complete absorption and wide distribution in vivo [9, 10]. At present, TMP has been used in the clinical treatment of stroke, asthma, emphysema, cor pulmonale, chronic respiratory failure, adult respiratory distress syndrome, and other diseases [11]. Based on the above pharmacological activities, we boldly assumed that ligustrazine has a good repair effect on thin endometrium. This article focuses on the repair effect of ligustrazine on thin endometrium rats.

## 2. Materials and Methods

### 2.1. Animal

SPF female SD rats (8 weeks, 220~250 g), animal Certificate No. SCXK (Su) 2012-0007, were provided by the Experimental Center of Nantong University.

### 2.2. Experimental Drug

Ligustrazine (purity: 97%) was purchased from Tianjin Vientiane HengYuan Technology Co., Ltd.

### 2.3. Reagent

VEGF(#2463), VEGFR-2 (#9698), PI3K (#4249), and AKT (#4691) antibodies were purchased from Cell Signaling Technology (Danvers, USA).

### 2.4. Experimental Process

Sixty rats with normal estrous cycle were included in this experiment. The rats were randomly divided into a control group of 10 rats and an experimental group of 40 rats. In the experimental group, rats were modeled on thin endometrium of rats according to the method of Gao Hong et al., i.e., injecting anhydrous ethanol into the uterine cavity of female rats for 5 minutes. All rats were randomly divided into 5 groups with 12 rats in each group: (1) control group(C); (2) model group (M); (3) aspirin (AS, 6.75 mg/kg ); (4) ligustrazine (TMP, 20 mg/kg ) group; (5) ligustrazine (TMP, 40 mg/kg) group. Aspirin and TMP, respectively, dissolved the drug in 2ml of water for gastric administration.

### 2.5. Measurement of Endometrial Thickness

The rats were killed by neck fracture, and the uterus was removed quickly under relatively sterile conditions. The surrounding tissues were removed and the uterus was dissected. The thickness of the endometrium was measured by Leica Qwerp image processing software.

### 2.6. HE Staining of Endometrium

Uterus was taken and fixed with paraformaldehyde, embedded, and sectioned. Then the endometrial stroma, blood vessels, epithelial cells, and glands were observed with HE staining histology.

### 2.7. RT-PCR Detection of VEGF, VEGFR, PI3K, and AKT mRNA in Endometrial Tissues

Rat uterus tissues of each group were collected, and the total mRNA of endometrial tissue was extracted according to the instructions of the kit. The mRNA levels of PI3K, AKT, VEGF, and VEGFR-2 in endometrial tissue were detected by RT-PCR. The primer sequences are shown in Table 1. According to the manufacturer's instructions, the total RNA in the endometrium was extracted from the endometrium tissue using TRIZOL reagent (Invitrogen, California Life Technology Company, USA ). RNA purity was measured with 2000 nm thermal science drops (Massachusetts, USA). Next, RNA was transcribed into cDNA using a reverse transcriptase kit (Takara Biotechnology Company, Kyoto, Japan). Quantitative real-time PCR (qRT-PCR) analysis was performed using ChamQ SYBR qPCR main mixture (China Nanjing Vazyme Biotechnology Co., Ltd) and CFX management software (Bio-Rad Laboratory Co., Ltd). Standardized expression of GAPDH was analyzed in each sample. The mRNA relative levels of PI3K, AKT, VEGF, and VEGFR-2 were analyzed by 2^−ΔΔCt^.

### 2.8. Western Blotting Detection of the Protein Levels of VEGF, VEGFR-2, PI3K, and AKT

Rat uterus tissues were collected and the total protein in the endometrium tissues was extracted according to the instructions of the kit. Western blotting technique was used to detect the levels of VEGF, VEGFR-2, PI3K, and AKT in endometrial tissue. Liquid nitrogen grinds endometrium tissue to the state of single cell, adding splitting solution, splitting on ice for 30 min, centrifuge at 12000× g 4°C for 15 min to obtain supernatant. BCA method was used to measure the protein concentration, then buffer was added to adjust the protein concentration, and polyacrylamide gel electrophoresis (SDS-PAGE) was performed. After electrophoresis, the protein was transferred to polyvinylidene fluoride (PVDF) membrane and sealed with 5% skimmed milk powder for 60 min. PVDF membranes were placed in VEGF(#2463, 1:1000), VEGFR-2 (#9698, 1:1000), PI3K (#4249,1:1000), and AKT (#4691,1:1000) solution, incubated overnight at 4°C, washed with TBST 3 times, incubated with HRP labeled second antibody (1:10000) for 2 h, and washed with TBST 3 times. The gray value of each strip was analyzed by exposing and scanning the strip using a gel imaging system.

### 2.9. Statistical Analysis

The experimental data were statistically processed by graph pad prism 7.0 biostatistical software, the measurement data were expressed mean ± standard deviation, one-way ANOVA was performed with statistical software, and p < 0.05 indicated that the difference was statistically significant.

## 3. Results

### 3.1. General Observation

During the experiment, all rats were in good living condition and showed no obvious difference in daily conditions. No obvious adverse drug reactions or other discomfort occurred after administration.

### 3.2. Effect of Ligustrazine on Endometrial Thickness

**Figure 1:**
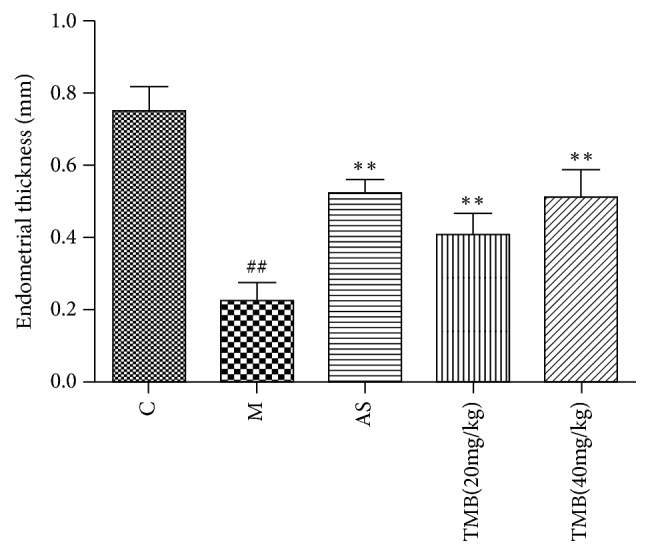
*Effect of Ligustrazine on endometrial thickness*. All values given are the mean ± SD. ^#^P<0.05 and ^##^P<0.01 vs. control group. ^*∗*^P<0.05 and ^*∗∗*^P<0.01 vs. M group.

The thickness of endometrium is the vertical distance from the junction of endometrium and myometrium to uterine cavity measured by Leica Qwerp image processing software. As shown in Figure 1, compared with the control group, the thickness of endometrium in the model group was significantly thinner. Compared with the model group, ligustrazine and aspirin could significantly increase the endometrial thickness of rats.

### 3.3. Effect of Ligustrazine on Morphology of Endometrium

**Figure 2:**
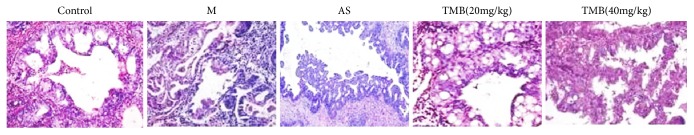
*Effect of Ligustrazine on morphology of endometrium(x200)*. All values given are the mean ± SD. ^#^P<0.05 and ^##^P<0.01 vs. control group. ^*∗*^P<0.05 and ^*∗∗*^P<0.01 vs. M group.

Compared with the control group, the model group had thinner endometrium, fewer glands, loose arrangement, intact glandular epithelial cells, a single layer of cubic epithelium, dense stroma, fewer cells and smaller cells, and sparse vascular distribution in most areas. The thickness of intrauterine membrane and the number of glandular bodies in the large group of ligustrazine and aspirin groups were significantly increased compared with those in the model group, and the interstitial blood tubes were divided into density sets, as shown in Figure 2.

### 3.4. Effects of Ligustrazine on mRNA Levels of VEGF, VEGFR-2, PI3K, and AKT in Endometrial Tissues

**Figure 3:**
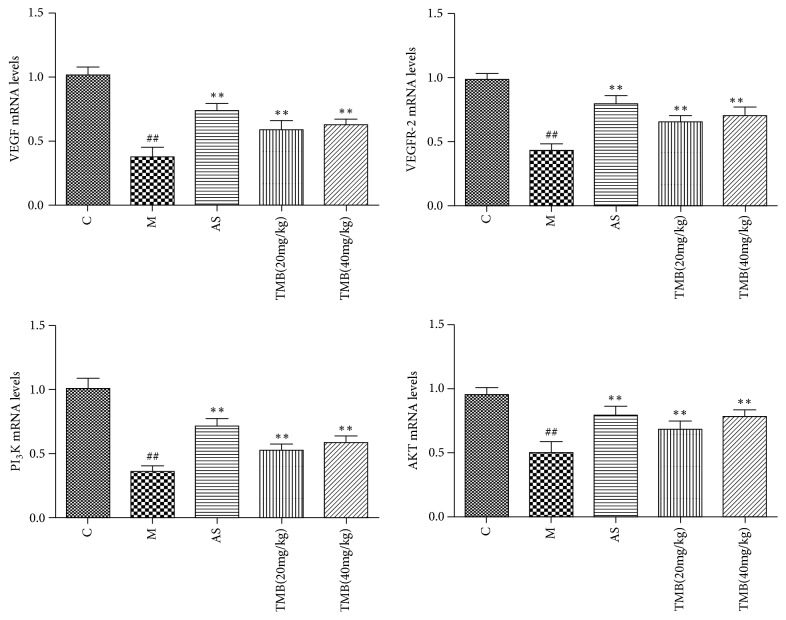
*Effects of Ligustrazine on mRNA expression of VEGF, VEGFR-2, PI3K, and AKT in endometrial tissues*. All values given are the mean ± SD. ^#^P<0.05 and ^##^P<0.01 vs. control group. ^*∗*^P<0.05 and ^*∗∗*^P<0.01 vs. M group.

RT-PCR results showed that the mRNA* levels* of VEGF, VEGFR-2, PI3K, and AKT in the model group were significantly lower than those in the control group. The mRNA* levels* of VEGF, VEGFR-2, PI3K, and AKT in ligustrazine and aspirin groups were significantly higher than those in model group (Figure 3).

### 3.5. Effects of Ligustrazine on Protein Levels of VEGF, VEGFR-2, PI3K, and AKT in Endometrial Tissues

**Figure 4:**
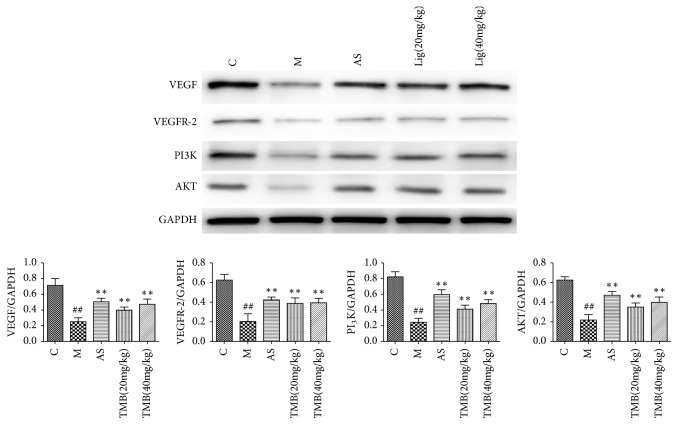
*Effects of Ligustrazine on protein expression of VEGF, VEGFR-2, PI3K, and AKT in endometrial tissues*. All values given are the mean ± SD. ^#^P<0.05 and ^##^P<0.01 vs. control group. ^*∗*^P<0.05 and ^*∗∗*^P<0.01 vs. M group.

Western Blotting results showed that the protein* levels* of VEGF, VEGFR-2, PI3K, and AKT in the model group were significantly lower than those in the control group. The protein* levels* of VEGF, VEGFR-2, PI3K, and AKT in ligustrazine and aspirin groups were significantly higher than those in model group (Figure 4).

## 4. Discussion

The successful implantation of an embryo depends on a certain thickness of the intrauterine membrane to provide nutrition, which is the guarantee of a good pregnancy outcome [12, 13]. Compared with the thicker endometrium, the clinical pregnancy rate and the continued pregnancy rate decreased significantly when the thickness of endometrium after ovulation was less than 7-8 mm, which was confirmed by a large amount of clinical research [14, 15]. At the end of the implantation window, the thickness of the endometrium needs to reach before it can be implanted by inducing embryo vesicles. At present, the boundary of this body number value is not uniform. Many mathematicians believe that the thickness of endometrium is as low as > 7 mm, which is the antecedent of pregnancy, when the thickness of endometrium is <6 mm making it difficult to get pregnant [16, 17]. In recent years, domestic researchers have also tried to increase the thickness of endometrium by various methods in order to achieve the goal of increasing the pregnancy rate and achieve success. This proves that the thickness of endometrium is one of the key factors affecting the outcome of assisted reproductive technology.

At present, the mechanism of thin endometrium is still unclear, and there is no unified diagnostic standard because there has not been a standardized treatment plan for the temporary bed in recent years.

However, Mina [18, 19] and others have studied the factors related to the blood flow and the growth of the inner membrane of the thin uterus and found the common characteristics of the thin inner membrane: the increase of the blood flow resistance of the inner membrane and the decrease of the blood supply lead to the damage of the development of glandular epithelial cells and the growth of the inner membrane of the blood tube, (VEGF) expression decreased, which resulted in poor vascular growth in the endometrium, which in turn affected the blood flow in the endometrium. The process was repeated repeatedly, and the most end-result was that the growth of the inner membrane was limited by thin endometrium [20]. The reason why the increase of intimal resistance is an initial factor is unknown, but after administration of drugs to improve blood flow, the resistance of intimal blood flow drops. The upregulation of VEGF expression and the thickening of endometrium prove that lack of blood and decrease of blood supply are one of the reasons for thin endometrium. Vascular endothelial growth factor (VEGF), also known as vascular permeability factor, can induce the formation of blood vessels and affect vascular permeability [21]. The signal cascade mediated by VEGF and its receptor protein tyrosine kinase (especially VEGFR-2) is an important mediation pathway. VEGF/VEGFR-2 mediated signal cascade pathway can control endothelial cell (EPC) proliferation, migration, survival, and permeability changes, thus promoting angiogenesis. The secretion of VEGF and VEGFR-2 can enhance angiogenesis in endometriosis. VEGF can play a different role by binding tyrosine kinase receptors on the cell surface to activate the corresponding signal pathways, while phosphatidylinositol 3-kinase (PI3K) exists on the cell membrane and can be activated by many receptor tyrosine kinases. VEGF continuously stimulates angiogenesis by activating phosphatidylinositol-3 killase (PI3K)/serine-threonine protein kinase (AKT) [22]. Angiogenesis is an important condition for endometrial growth, and vascular endothelial growth factor (VEGF) is the main inducer of angiogenesis and can promote angiogenesis. In this study, we detected the expression of VEGF, VEGFR-2, PI3K, and AKT mRNA and protein in intima. The results showed that the levels of VEGF and VEGFR-2 mRNA and protein in rat endometrium were significantly upregulated in the ligustrazine and aspirin groups than in the model group and blank group. The expression trends of PI3K and AKT are similar to those of VEGF and VEGF-2. It has been proved above that ligustrazine can promote endometrial hyperplasia, and VEGF and VEGFR-2 have strong vascular regeneration and repair effects. The high expression of VEGF and VEGFR-2 confirmed that the thickening of thin endometrium can be achieved through the regeneration and repair of blood vessels. The upregulation of PI3K and AKT suggests that the regeneration and repair of thin endometrium is achieved through PI3K/AKT signaling pathway. Therefore, the above results may suggest that ligustrazine activates PI3K/AKT signaling pathway through upregulation of VEGF and VEGFR-2 expression to induce the repair of thin endometrium in rats.

Ligustrazine could improve the pathological changes of endometrium, promote the proliferation of endometrium, and upregulate the levels of VEGF, VEGFR-2, PI3K, and AKT in rat endometrium. VEGF and VEGFR-2 are closely related to vascular regeneration and tissue repair, and their high expression can promote the repair of thin endometrium, suggesting that ligustrazine activates PI3K/Akt signaling pathway by upregulating VEGF and VEGFR-2 levels, thus inducing the repair of thin endometrium in rats.

## Figures and Tables

**Table 1:** Primers used in this study.

Genes	Primer sequence (5′-3′)	Primer sequence (3′-5′)
GAPDH	CTGAGGACCAGGTTGTCTCC	GAGGGCCTCTCTCTTGCTCT
VEGF	AAATCCTGGAGCGTTCACTGT	TTCGTTTAACTCAAGCTCCTC
VEGFR-2	CCTGGCTATGAAGGAAGATGG	ACTGGAGTATTTCCGTGACC
PI3K	TATTCCAGACGCATTTCCAC	ATTCAGCCATTCATTCCACC
AKT	TCTAGGCGTGAGATTGTG	CTTAATGTGCCCGTCCTGT

## Data Availability

The data used to support the findings of this study are available from the corresponding author upon request.
